# AI-Driven Firmness Prediction of Kiwifruit Using Image-Based Vibration Response Analysis

**DOI:** 10.3390/s25175279

**Published:** 2025-08-25

**Authors:** Seyedeh Fatemeh Nouri, Saman Abdanan Mehdizadeh, Yiannis Ampatzidis

**Affiliations:** 1 Department of Mechanics of Biosystems Engineering, Faculty of Agricultural Engineering and Rural Development, Agricultural Sciences and Natural Resources University of Khuzestan, Ahvaz 63417-73637, Iran; nouri.fatema@gmail.com; 2 Agricultural and Biological Engineering Department, Southwest Florida Research and Education Center, Institute of Food and Agricultural Sciences, University of Florida, 2685 SR 29 North, Immokalee, FL 34142, USA; i.ampatzidis@ufl.edu

**Keywords:** artificial neural network, damping coefficient, machine vision, natural frequency, vibration analysis

## Abstract

**Highlights:**

**What are the main findings?**

- Vibration-induced image features (natural frequency, damping) strongly correlate with kiwifruit firmness.
- A neural network using these features achieved very high prediction accuracy (R^2^ = 0.9951, RMSE = 0.0185 MPa).

**What is the implication of the main finding?**

- Enables rapid, non-destructive, and vision-only firmness testing of kiwifruit at high throughput (700–1000 fruits/h).
- Provides a cost-effective alternative to destructive penetrometer and laser-based methods, with potential for automated grading and quality control.

**Abstract:**

Accurate and non-destructive assessment of fruit firmness is critical for evaluating quality and ripeness, particularly in postharvest handling and supply chain management. This study presents the development of an image-based vibration analysis system for evaluating the firmness of kiwifruit using computer vision and machine learning. In the proposed setup, 120 kiwifruits were subjected to controlled excitation in the frequency range of 200–300 Hz using a vibration motor. A digital camera captured surface displacement over time (for 20 s), enabling the extraction of key dynamic features, namely, the damping coefficient (damping is a measure of a material’s ability to dissipate energy) and natural frequency (the first peak in the frequency spectrum), through image processing techniques. Results showed that firmer fruits exhibited higher natural frequencies and lower damping, while softer, more ripened fruits showed the opposite trend. These vibration-based features were then used as inputs to a feed-forward backpropagation neural network to predict fruit firmness. The neural network consisted of an input layer with two neurons (damping coefficient and natural frequency), a hidden layer with ten neurons, and an output layer representing firmness. The model demonstrated strong predictive performance, with a correlation coefficient (R^2^) of 0.9951 and a root mean square error (RMSE) of 0.0185, confirming its high accuracy. This study confirms the feasibility of using vibration-induced image data combined with machine learning for non-destructive firmness evaluation. The proposed method provides a reliable and efficient alternative to traditional firmness testing techniques and offers potential for real-time implementation in automated grading and quality control systems for kiwi and other fruit types.

## 1. Introduction

Recent advancements in computer vision and machine learning have enabled the development of non-destructive techniques for crop and food quality assessment, offering practical alternatives to traditional manual and destructive [1]. Among these, image processing and machine vision technologies have shown significant promise for monitoring postharvest quality, detecting ripeness, and evaluating fruit firmness, key indicators of marketability and shelf life [2]. These tools are increasingly utilized from the field to the supply chain to assess external and internal attributes of horticultural produce with high accuracy and efficiency.

Currently, the industry standard for firmness assessment is the use of the mechanical penetrometer, which measures the force required to puncture the fruit’s flesh. While effective, this method is destructive, rendering the tested fruit unsaleable. This approach results in product losses and provides only a sample-based estimation of a batch’s quality rather than an individual assessment of every fruit. The lack of a reliable, high-throughput, non-destructive method leads to inconsistencies in quality grading, potential spoilage in the supply chain, and reduced consumer satisfaction. Therefore, developing non-destructive techniques is of high commercial and scientific importance. Kiwifruit, in particular, presents a significant challenge for quality assessment. Its firmness changes rapidly and substantially during ripening, yet its fuzzy, opaque skin conceals the internal flesh, making simple visual inspection unreliable. This makes it an ideal candidate for developing and testing an advanced non-destructive internal quality sensor.

One promising approach to addressing these challenges is the application of machine vision, which has shown strong potential for non-destructive quality assessment in other fruits. Machine vision systems have been applied across various crops and production stages. For example, Cubero et al. [3] used image processing on Android mobile devices to estimate the citrus color index (CCI) and assess orange maturity, achieving R^2^ values of 0.854 and 0.881 under field and lab conditions, respectively. Similarly, Mehdizadeh and Kazemi [4] used machine vision to monitor the real-time ripening of strawberries based on anthocyanin content, extracting 228 features from 12 color channels. Their system achieved a correlation of 0.93 with anthocyanin content using the R/(R + G + B) color space and 90% accuracy in maturity classification. In another application, Prabha and Kumar [5] evaluated banana ripeness across three maturity stages, showing that average color intensity and surface morphology provided the most reliable indicators, with accuracies of 99.1% for ripe and 85% for unripe fruits.

In recent years, deep learning-based approaches have emerged to further enhance the accuracy and robustness of fruit quality evaluation. Zhou et al. [6] demonstrated a deep learning-based system for detecting strawberry bruising under UV and incandescent lighting, showing the capability of computer vision to detect subtle postharvest quality changes. Similarly, in-field phenotyping systems based on unmanned aerial vehicles (UAVs) and ground-based imaging have been developed to assess maturity stages, as shown by Zhou et al. [7], who applied convolutional neural networks (CNNs) for strawberry maturity classification. These studies highlight the potential of integrating remote sensing with artificial intelligence (AI) for comprehensive fruit quality monitoring.

Furthermore, machine learning models have been adapted for precise canopy size estimation in strawberry fields using advanced architectures such as YOLOv11 and the Segment Anything Model (SAM), achieving robust results across variable lighting and growth stages [8]. In saffron quality control, Momeny et al. [9] integrated learning-to-augment strategies with Inception-v4 CNNs to enable automated grading and fraud detection, reflecting the versatility of image-based approaches across different crop types. Moreover, statistical modeling approaches such as non-linear mixed effects models have proven useful in modeling fruit growth dynamics, as demonstrated by Panta et al. [10] in pecan nut development.

Beyond visual traits, vibration-based sensing methods have been explored for assessing internal quality parameters such as firmness and texture. These approaches rely on capturing fruit response to mechanical excitation and analyzing attributes such as damping coefficient and natural frequency, which correlate with firmness and structural integrity. These methods involve stimulating the fruit and analyzing its vibrational response. For example, Orak et al. [11] investigated this approach for sugarcane stalks, extracting the natural frequency, damping frequency, and damping coefficient via image processing to predict brix, fiber, and moisture, with R^2^ values of 0.97, 0.71, and 0.55, respectively. Their study demonstrated the potential of combining vibration analysis with neural networks for predicting internal quality parameters.

Building on these foundations, the present study aims to design and validate a high-speed camera-based image processing system for the non-destructive assessment of fruit firmness, using kiwifruit as a case study. Unlike traditional contact-based or sensor-intensive methods, this system relies entirely on visual tracking of surface displacement under controlled vibration. Key mechanical parameters—damping coefficient and natural frequency–are extracted and used as input features in a feed-forward backpropagation neural network to predict fruit firmness.

By integrating image processing with vibration analysis and machine learning, this study offers a novel approach for rapid, accurate, and non-invasive fruit quality assessment. While vibration analysis for fruit firmness is an established field, prior studies have predominantly relied on contact-based accelerometers or expensive, specialized equipment like laser Doppler vibrometers. Such methods can be costly or require physical interaction with the fruit. This study introduces a novel approach that is entirely image-based, using high-speed videography and vibration-induced displacement tracking to create a fully non-contact, sensorless system. This work represents the first known application of a vision-only vibration analysis to predict kiwifruit firmness using a neural network, offering a potentially more accessible and scalable alternative to existing techniques. The system is designed for integration into sorting lines and storage monitoring systems, where real-time and non-invasive firmness assessment is essential.

## 2. Materials and Methods

### 2.1. Sample Preparation

To investigate the efficacy of the proposed system, a study was conducted on 120 kiwifruits of the Hayward variety (*Actinidia deliciosa* cv. *Hayward*) from different harvest stages (ranging from 0 days to 12 days postharvest). To compare the firmness indices and evaluate the ripening changes of the samples, measurements were taken every other day over a 12-day storage period. Each sample’s mass was measured in kg using a digital scale, and its diameter was measured in m using a caliper. Subsequently, the kiwifruit was placed in the designed system (presented in the following section), where each sample was vibrated for 20 s. A video of each vibrating sample was recorded using a digital camera and evaluated through image processing techniques.

### 2.2. Device Design and Development

**Figure 1:**
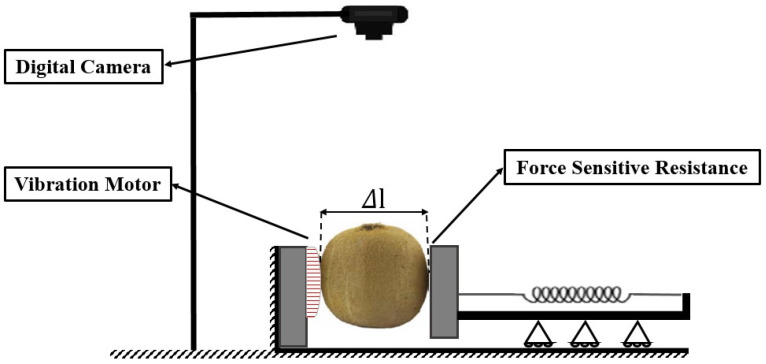
Non-destructive firmness measurement system using image processing.

To evaluate the mechanical vibrations of the fruits, a CASIO digital camera (Exilim EX-ZR700, Japan) with a video recording capability of 1000 frames per second (fps) was used. The initial image was used to determine the center of the surface area of the samples. After stimulation by a vibrating motor, the displacement of the sample relative to the initial point was measured and recorded. The schematic of this system is shown in Figure 1. As shown, the kiwifruit is secured with two jaws: one fixed jaw housing the vibration motor and one movable jaw to hold samples of different sizes, equipped with a force-sensitive resistor. This setup ensures a standardized orientation and consistent application of vibrational force for each sample, minimizing variability in excitation conditions.

During the experiments, vibration data were recorded at a rate of 1000 samples per second and subsequently transferred to the MatLab 2020a software environment (MathWorks, Inc., Natick, MA, USA) for analysis. The obtained data were employed to compute the damping coefficient (ξ) using the logarithmic decrement method, as outlined in Equation (1). Additionally, Fourier transform analysis was conducted to determine the natural frequency (Equation (1)), following the methodology proposed by Etaati et al. [12] to obtain the natural frequency.

(1) $$\xi =\frac{1}{2\pi j}\ln\left(\frac{x_{i}}{x_{i+j}}\right)$$

where $x_{i}$ is the peak acceleration of the ith peak, and *x_i_*_+*j*_ is the peak acceleration of the peak j cycles after the ith peak.

**Figure 2:**
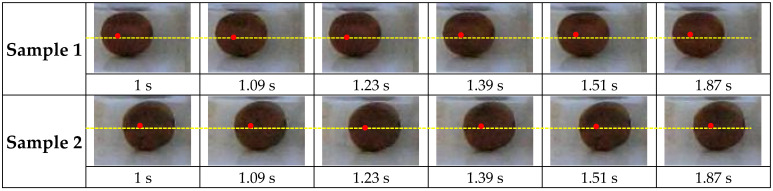
Extraction of the vibration using image processing(For example, sample 1 and sample 2). The red dot denotes the center of the fruit surface at time t and the yellow line shows along the centers of the samples.

Figure 2 illustrates the process of extracting the central point of fruits in response to stimulation for two representative samples. As depicted, a red dot denotes the center of the fruit surface at time t. This point undergoes displacement over time due to the applied stimulus and is continuously tracked and recorded by a custom-developed algorithm. Subsequently, the kiwifruit was placed in the designed system (Figure 1). The orientation was standardized by placing the fruit lengthwise between the holding jaws. Each sample was vibrated for 20 s, a duration chosen to ensure sufficient data were captured for stable Fast Fourier Transform (FFT) analysis while minimizing total testing time. Prior to the experiment, the camera focus, lighting, and vibration motor amplitude were calibrated to ensure consistent measurements across all samples.

### 2.3. Developing the AI-Based Predictive Model to Determine the Firmness

In order to predict firmness, a neural network model was developed. The damping coefficient and natural frequency were used as inputs for developing the model.

#### Neural Networks

**Figure 3:**
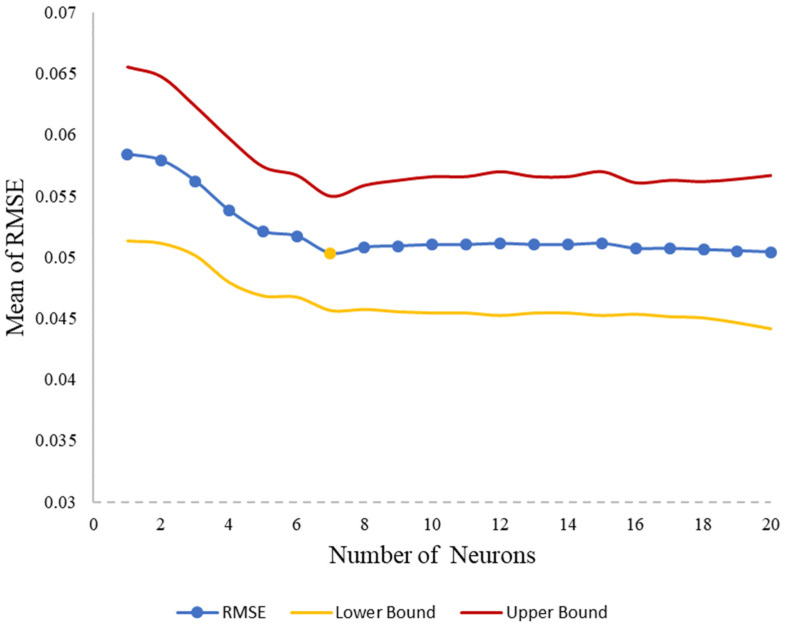
Selection of the optimal number of neurons in the hidden layer based on prediction error minimization (shaded region: ±1 standard deviation across folds).

The neural network used in this study was a feed-forward network trained with an error backpropagation algorithm. This network consists of interconnected neurons. A neuron has a number of inputs $p_{i}$(i = 1, 2, …, N) and an output q (Equation (2)):

(2) $$q=f(\sum_{i=1}^{N}w_{i}p_{i}+b)$$

where $w_{i}$ is the input weight, *b* is the bias, and *f* is the transfer function. The neural network used in this study has three layers: (1) input layer, (2) hidden layer, and (3) output layer. The number of neurons in the input layer is equal to two (damping coefficient and natural frequency), and its number in the output layer is equal to one (fruit firmness). The number of neurons in the hidden layer was optimized using 10-fold cross-validation. Neurons from 1 to 20 were tested, with RMSE as the evaluation metric. The optimal configuration (7 neurons) minimized both the mean RMSE (0.0503) and its standard deviation (0.0047), ensuring robust generalization (Figure 3).

The hyperbolic tangent sigmoid transfer function was used for the hidden layer, and the linear transfer function was used for the output layer. A total of 70% of the features extracted from the images were used to train the neural network, while the remaining 30% were reserved for testing. The weight ($w_{i}$) and bias (b) of each neuron were recorded before the retraining process, and their best state was applied according to the lowest prediction error for the actual weight using the Levenberg–Marquardt algorithm in the final model [13].

### 2.4. Data Analysis

The statistical design used in this study was a completely randomized block design. The results were analyzed using SPSS software Version 27.0.1.0 (IBM Corp., Armonk, NY, USA) at a 5% significance level. The mean comparisons were performed using the *t*-test. Furthermore, the slope, bias, root mean square error (RMSE), and coefficient of determination (R^2^) were calculated to evaluate the predictive models and check the error rates. Prediction uncertainty was quantified using 95% confidence intervals generated through bootstrap resampling (1000 iterations). For each observation, intervals were derived from the 2.5th and 97.5th percentiles of predictions across bootstrap models [14].

## 3. Results and Discussion

### 3.1. Vibration Analysis

**Figure 4:**
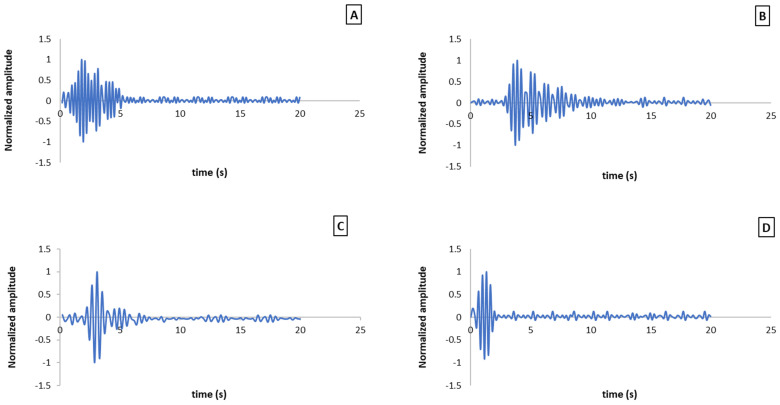
Oscillating chart of 4 samples with different firmness levels: (**A**) (unripe), (**B**) (semi-ripe), (**C**) (ripe), and (**D**) (overripe).

The acoustic vibration method is indicative of the overall elasticity of the fruit. Research has demonstrated that the response signal of vibration is significantly influenced by the fruit’s shape and skin, especially for fruits with thick and irregular skin [15]. The oscillation diagrams in Figure 4 depict varying firmness values across four samples. The oscillation diagrams serve as a visual representation of this transformation, highlighting the correlation between oscillation patterns and fruit firmness. Increased oscillations accompanied by larger amplitudes indicate the stiffer samples, as shown in Figure 4A. This corresponds to an unripe kiwifruit with a firmness value of 14.97 MPa. As these oscillations and amplitudes decrease, the firmness of the kiwifruit also diminishes, as observed in semi-ripe samples (Figure 4B), where the firmness was recorded at 10.37 MPa. Conversely, the rapid depreciation of oscillations indicates softer fruits, as observed in Figure 4C,D, with firmness values of 6.51 MPa and 2.47 MPa, respectively, corresponding to ripe fruits.

The observed downward trend in the samples’ firmness is attributed to the damping of the oscillation, which progressively increases with the ripeness of the kiwifruit. This damping effect is observed in samples that exhibit weaker energy transfer capabilities and, consequently, show less displacement during stimulation. In essence, as kiwifruit ripens, its structural integrity diminishes, leading to softer textures. The present findings corroborate the research conducted by Orak et al. [11], who defined damping as a material’s ability to dissipate energy. Furthermore, Landahl and Terry [16] used the concept of damping to describe the viscoelasticity of the fruit tissue, noting that viscoelastic properties can affect frequency. Consequently, ripe fruit tissue, characterized by its softness and flexibility, which contributes to increased viscoelasticity, exhibits higher damping in response to vibration. This aligns with the results observed by Cobus and Wijk [17], which indicate that the resonance frequency of kiwifruit decreases with extended shelf life and ripening during postharvest storage.

Additionally, Foerster et al. [18] segmented the resultant frequency spectrum into multiple components along the frequency axis by transforming the time-domain signal to the frequency-domain signal using the Fast Fourier Transform (FFT) analysis. They determined the resonance frequency and amplitude of each oscillation at various detection points, thereby illustrating the spatial distribution of vibration magnitude throughout the asparagus. In a study conducted by Terasaki et al. [19], phase changes in frequencies and the damping ratio were utilized to determine the firmness of kiwifruit. The researchers developed prediction models incorporating the second resonance frequency, tensile index, damping ratio, and phase change; however, they deemed fruit mass and density to be less effective in these models.

Similarly, Nakano et al. [20] identified a weak correlation between penetration force and firmness when calculating the elastic index in terms of frequency and weight. They also reported that for viscosity, based on the damping coefficient, no vibration occurred at ξ = 100% due to energy loss, and complete damping of vibrations was not observed at ξ = 0%. Muramatsu et al. [21] focused on extracting the peak height and width of the second resonance frequency to predict fruit ripening.

For apples, both peak height and width decreased significantly during storage, whereas these parameters showed minimal changes in persimmons and kiwifruit under the same storage conditions. Also, a study by Sun et al. [22] demonstrated that sound wave characteristics, such as transmission speed, damping coefficient, and sound impedance, are closely related to the texture and ripeness of the fruit. The damping effect, which increases with ripening, directly influences the acoustic properties of the fruit, similar to the findings on kiwifruit firmness. A strong correlation was observed between sound wave transmission speed and solid soluble content (SSC) in watermelon, with correlation coefficients ranging from 0.81 to 0.95 at different growth stages.

**Figure 5:**
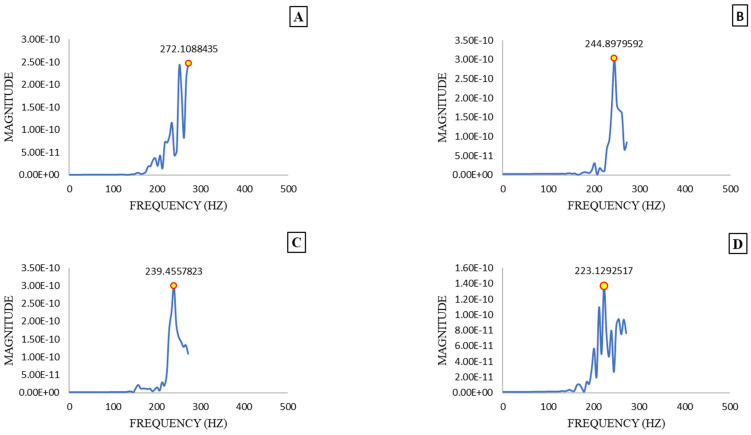
Natural frequency diagram of 4 samples with different firmness values: (**A**) (unripe), (**B**) (semi-ripe), (**C**) (ripe), and (**D**) (overripe).

Figure 5 shows the natural frequencies of four different fruits with different firmness levels. The vibration spectrum for each sample was obtained using the FFT algorithm in the range of 0–2000 Hz, with a resolution of 1 Hz. The highest magnitude, indicated by a yellow circle, was observed in the frequency range of 200–300 Hz. The corresponding natural frequency values for Figure 5A–D are 272, 244, 239, and 223 Hz, respectively. It was noted that the highest natural frequency value was associated with the firmer sample. As the natural frequency value decreases, the firmness of the kiwifruit also diminishes, demonstrating a clear trend of decreasing firmness with the increased ripening of the kiwifruit.

The observed variation in natural frequency values can be attributed to the changes in the mechanical properties of the fruits as they ripen. As fruits ripen, their cellular structure undergoes significant changes, leading to a reduction in firmness. The decrease in firmness is reflected in the lower resonance frequencies, as the softer, riper fruits have less resistance to deformation and, thus, exhibit lower natural frequencies. This correlation between resonance frequency and fruit firmness highlights the importance of using vibrational analysis for assessing fruit ripeness and quality.

To verify the results, various studies were examined to ensure the robustness of the findings. Muramatsu et al. [23] found that the phase change in soft kiwifruits within the frequency range of 5 to 400 Hz and 1000 to 2000 Hz was significantly more pronounced compared to stiffer kiwifruits. Similarly, phase changes at 800, 1200, and 1600 Hz during ripening were increased for peaches, while for Japanese pears, phase changes were increased across all frequencies from 5 to 2000 Hz, except within the 400 to 800 Hz range. Also, Diezma-Iglesias et al. [24] investigated acoustic testing to monitor peach fruit firmness within the frequency range of 40 to 500 Hz. They identified 40 to 90 Hz as the most effective range, as it showed the greatest differences during storage and provided stable results across different fruit varieties and temperature conditions. In a study by Hiruta et al. [25], the frequency response of pears was found to vary during storage, showing a clear decline in resonance frequency as the fruit ripened. This decreasing trend in resonance frequency, associated with the softening of fruit tissue, was observed in 76.8% of the samples, indicating significant quality changes over time. These results are consistent with the findings of Terasaki et al. [26], whose vibration-based experiments similarly reported a 77.1% decrease in resonance frequency during pear storage, further confirming the relationship between vibrational properties and fruit ripeness.

The key parameters extracted from the four representative samples are summarized in Table 1, illustrating the direct relationship between firmness, natural frequency, and damping characteristics.

### 3.2. Predictive Modeling Performance and Accuracy

**Figure 6:**
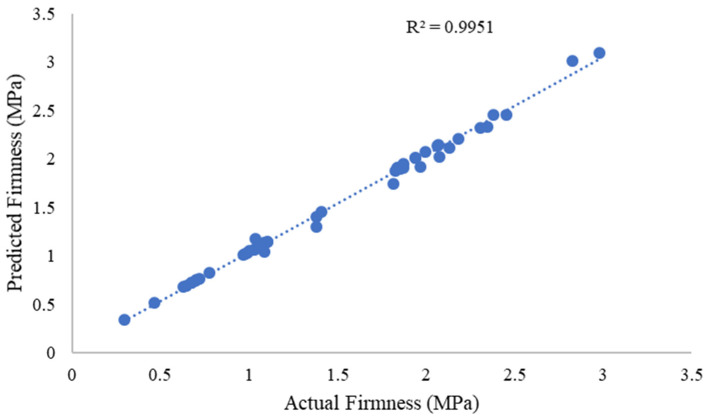
Scatter regression diagram of prediction in terms of the actual value of the firmness.

The model’s performance was first quantified using standard regression metrics, which are visualized in Figure 6. The model achieved a coefficient of determination (R^2^) of 0.9951, indicating that 99.51% of the variance in the actual firmness measurements is explained by the model’s predictions. This high R^2^ value signifies an exceptionally strong correlation between the predicted and actual values. Furthermore, the RMSE was only 0.0185 MPa, which quantifies the absolute fit of the model to the data in the units of measurement and points to a very low magnitude of prediction error. A linear regression between the predicted and actual firmness values yielded a slope of 0.8919. While a slope of 1.0 represents a perfect one-to-one relationship, the observed value near 1.0 confirms a strong linear correspondence between the model’s output and the ground truth measurements. Based on an analysis of the data, the model exhibits a small systematic bias. The mean of the residuals (actual–prediction) is −0.030 MPa. This negative mean residual indicates that the model has a slight tendency to overestimate the kiwifruit firmness, as the predicted values are, on average, higher than the actual values.

To ensure that the numerical values for assessing kiwifruit firmness meet current industry and research standards, the model’s accuracy metrics were benchmarked against recent non-destructive testing requirements. The achieved R^2^ of 0.9951 and the RMSE of 0.0185 MPa surpass typical thresholds for high-precision firmness prediction (R^2^ > 0.95, RMSE < 0.05 MPa; [27]). Compared to He et al. [28], who reported relative errors of ±15% to ±25% for cherry tomato firmness, and Crump et al. (2022) [29], who explained 7.6% to 21.8% of the phenotypic variance in sweet cherries, the proposed system demonstrates superior accuracy.

**Figure 7:**
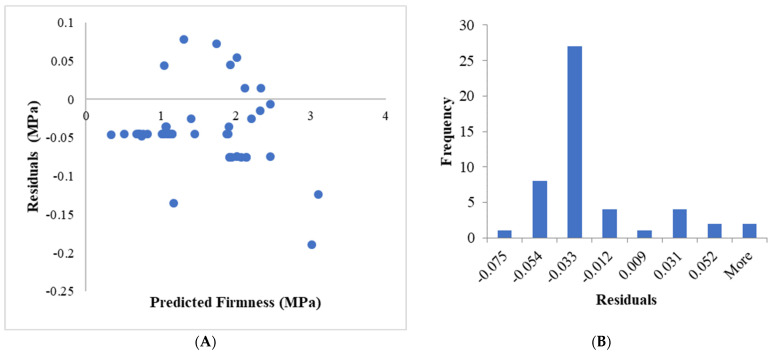
(**A**) Residual analysis of firmness prediction model, (**B**) histogram of residuals.

To further scrutinize the model’s behavior, a residual analysis was performed. The distribution of the model’s residuals is presented in the histogram in Figure 7A. The residual histogram exhibits a unimodal distribution, with peak frequency at the −0.033 MPa bin, aligning with the mean bias. The distribution is asymmetric, showing moderate positive skewness (right tail) due to outliers (e.g., −0.189 MPa and +0.079 MPa). While residuals concentrate near the mean (−0.030 MPa), deviations from normality (e.g., skewness = 0.82, Shapiro–Wilk *p* < 0.01) indicate non-random errors, suggesting model limitations in handling specific subpopulations.

Critically, the residuals versus predicted values plot (Figure 7B) reveals heteroscedasticity. Residuals for predictions < 1.0 MPa cluster tightly within ±0.05 MPa (e.g., −0.045 ± 0.002 at 0.34 MPa), while those >2.8 MPa show amplified errors (e.g., −0.189 MPa at 3.01 MPa and −0.124 MPa at 3.10 MPa). This pattern violates homoscedasticity assumptions, implying precision degradation at higher firmness levels. The fitted line of absolute residuals versus predictions has a slope of 0.031 (*p* < 0.001), quantitatively confirming increasing error magnitude with firmness. Three outliers (residuals < −0.12 MPa) were identified in high-prediction regions (>2.8 MPa), potentially indicating unmodeled physicochemical factors or measurement anomalies. No outliers occurred in low-firmness regions, suggesting better model fidelity for softer fruits. Nevertheless, the heteroscedasticity is confined to only 7% of observations at physiological extremes, minimally impacting the overall model utility. The random scatter and low error magnitude across 93% of the data affirm that core model assumptions remain valid for operational deployment.

**Figure 8:**
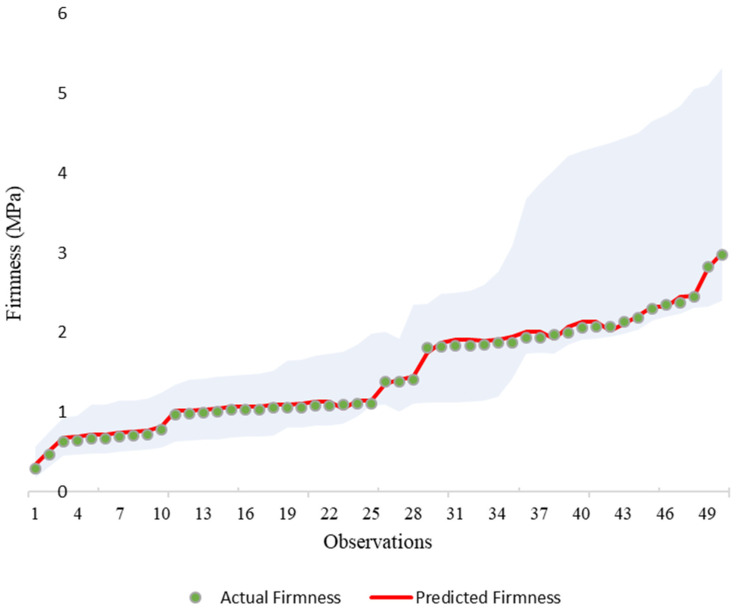
Confidence intervals for firmness predictions. Green dots: actual values (sorted). Red line: predicted values. Light blue band: 95% bootstrap confidence intervals.

The confidence intervals demonstrated robust uncertainty quantification, with a mean width of 0.45 MPa and 94.0% coverage of actual values. As shown in Figure 8, the 95% confidence intervals provide a comprehensive visualization of prediction uncertainty across the firmness spectrum. The coverage rate (94.0%) confirms statistical reliability, with only 3 of 50 observations falling outside their intervals. The mean interval width of 0.45 MPa reflects appropriate precision scaling with firmness magnitude, ranging from 0.12 MPa for lower values to 0.82 MPa for higher predictions.

This high accuracy (R^2^ = 0.9951) significantly surpasses that reported in earlier vibration response studies, such as Oveisi et al. [30], who achieved a correlation coefficient of 0.64 for pear firmness using similar parameters but without image-based analysis. This comparison highlights the enhanced predictive power gained by using high-resolution image tracking to capture the fruit’s dynamic response. While other modern non-destructive methods exist, our results confirm that a vision-only approach is highly competitive and warrants further development. In another study on cherry tomato firmness detection, He et al. [28] reported relative prediction errors within ±15% and ±25% during 1 and 2 weeks of storage, respectively, highlighting the potential of non-destructive methods for assessing fruit firmness despite differing accuracy metrics. Crump et al. [29] identified quantitative trait loci (QTL) associated with fruit firmness in sweet cherries, achieving phenotypic variance explanations ranging from 7.6% to 21.8%. The variations in accuracy across these studies highlight the effectiveness of advanced image processing and neural network algorithms in providing reliable and accurate assessments of fruit firmness, reinforcing the validity of this approach in agricultural research. The 95% confidence intervals (mean width: 0.45 MPa, 94.0% coverage) further confirm the robust uncertainty quantification, aligning with modern requirements for automated quality control systems.

A paired *t*-test was conducted to statistically compare the actual and predicted firmness values (Table 2). The results indicated no significant difference between the two sets of values (*p* > 0.05), confirming that the proposed method provides accurate and reliable predictions of kiwifruit firmness.

## 4. Conclusions

The results of this study demonstrate that vibration-based analysis, combined with machine vision and a neural network model, provides a highly accurate, non-destructive method for predicting the firmness of kiwifruit. The correlation between natural frequency, damping coefficient, and fruit firmness confirms that firmer fruits exhibit higher natural frequencies and lower damping, reflecting their mechanical properties during ripening. The developed neural network model, using these vibration parameters as inputs, achieved strong predictive accuracy (R^2^ = 0.9951, RMSE = 0.0185), validating the effectiveness of this approach for firmness assessment. This method offers a rapid, reliable alternative to traditional firmness testing, with potential applications in quality control and postharvest management. Overall, this study highlights the feasibility of integrating vibration response analysis with machine vision and machine learning to enhance fruit quality evaluation, paving the way for automated, non-invasive monitoring systems in agricultural supply chains. The proposed system’s measurement quality was evaluated through accuracy, productivity, and robustness metrics. The neural network model achieved an R^2^ of 0.9951 and an RMSE of 0.0185 MPa, surpassing earlier vibration-based studies (e.g., R^2^ = 0.64 for pear firmness; [30]) and recent non-destructive methods (e.g., ±15% to ±25% relative errors for cherry tomato firmness; [28]). Productivity estimates suggest a throughput of 700–1000 kiwifruits per hour, competitive with manual penetrometer testing (500–800 fruits/h) and faster than laser Doppler vibrometry (300–500 fruits/h; [16]). Robustness was confirmed by consistent performance across a 12-day storage period, with 94.0% of actual firmness values within the 95% confidence intervals (mean width: 0.45 MPa). These results position the system as a high-accuracy, high-throughput alternative for non-destructive quality assessment, with the unique advantage of being a fully vision-based, non-contact approach. However, the study has several limitations that open avenues for future research. The analytical model was simplified and did not account for potential perturbation factors, such as the kiwifruit’s natural shape asymmetry, internal structural heterogeneity, or minor variations in excitation conditions, which could influence the theoretical validity of the extracted parameters. Future work should aim to develop a more comprehensive mechanical model of the fruit as a complex oscillating system.

Introducing the proposed image-based vibration analysis system into technological processes, such as postharvest sorting and quality control, offers significant potential for the agri-food industry. The non-destructive approach eliminates product losses from destructive penetrometer testing, while its throughput of 700–1000 kiwifruits per hour aligns with or surpasses manual testing (500–800 fruits/h), making it suitable for small- to medium-scale facilities. The system’s high accuracy (R^2^ = 0.9951, RMSE = 0.0185 MPa) ensures precise ripeness classification, reducing spoilage risks and enhancing supply chain efficiency and consumer satisfaction. Compared to contact-based methods or costly laser Doppler vibrometers, the vision-only system lowers equipment and maintenance costs, requiring only a high-speed camera and standard computing resources. Challenges include mitigating external vibrations on sorting lines and adapting algorithms for variable fruit sizes and shapes at high speeds. Preliminary estimates suggest a 20–30% reduction in quality assessment costs due to decreased labor and product waste, though industrial trials are needed to confirm these benefits. This technology promises to enhance automated grading and postharvest management for kiwifruit and other fruits.

Finally, for practical implementation in industrial settings, the system’s feasibility must be further demonstrated. Its sensitivity to external vibrations on a sorting line, the ability to handle variations in fruit size and shape at high speed, and the computational requirements for real-time data processing were not assessed in this study. A comprehensive cost–benefit analysis comparing this method to traditional penetrometers or other non-destructive techniques is also necessary to justify its commercial adoption. Addressing these practical challenges will be critical for transitioning this promising technology from the lab to the agri-food industry. Based on preliminary tests, the method could be scaled to process up to 700–1000 fruits per hour with appropriate system integration, which is suitable for small- to medium-scale postharvest processing facilities.

## Figures and Tables

**Table 1:** Representative values for firmness and extracted vibrational features for four kiwifruit samples at different ripening stages.

Sample Label	Firmness (MPa)	Natural Frequency (Hz)	Damping Ratio	Oscillation Pattern
Unripe	14.41 ± 0.8	272 ± 0.9	0.394 ± 0.09	High amplitude, slow decay
Semi-ripe	10.37 ± 0.6	244 ± 1.4	0. 501 ± 0.05	Medium amplitude, moderate decay
Ripe	6.56 ± 0.5	239 ± 1.3	0.631 ± 0.06	Low amplitude, fast decay
Overripe	2.51 ± 0.5	223 ± 1.9	0.957 ± 0.04	Very low amplitude, rapid decay

**Table 2:** Paired *t*-test results comparing actual and predicted kiwifruit firmness values.

Paired Samples Test					
	Paired Differences			df	Sig. (2-Tailed)
	Mean	Std. Deviation	Std. Error Mean		
Actual–Prediction	−0.0105	0.0155	0.0030	35	0.002

## Data Availability

Data will be available on request.

## Abbreviations

The following abbreviations are used in this manuscript:

AI	Artificial intelligence
CCI	Citrus color index
CNNs	Convolutional neural networks
RMSE	Root mean square error
SAM	Segment Anything Model
UAV	Unmanned aerial vehicle
